# 

**Published:** 1899-04-15

**Authors:** 


**CADBURY'S COCOA** ABSOLUTELY PURE.

**CADBURY'S COCOA** NO DRUGS OR ALKALIES USED.

### THE HOSPITAL

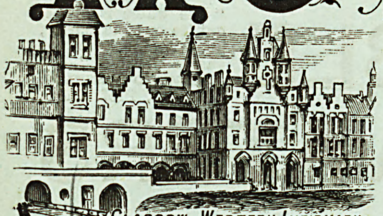

GLASGOW WESTERN INFIRMARY

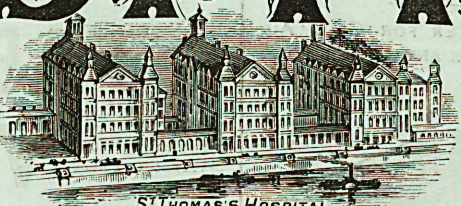

ST THOMAS'S HOSPITAL

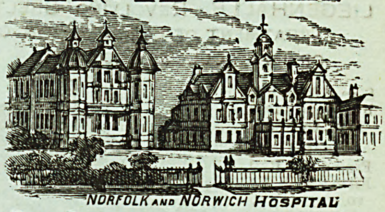

NORFOLK AND NORWICH HOSPITAL

No. 655. Vol. XXVI. [Registered as a Newspaper.] SATURDAY, APRIL 15TH, 1899. [Subscription Terms, see p. 38.] **PRICE TWOPENCE.**

### SPRING SPECIAL NUMBER.

